# Personality traits and cognition as predictors of long-term quality of life after transplantation for alcoholic liver disease

**DOI:** 10.1192/j.eurpsy.2024.636

**Published:** 2024-08-27

**Authors:** S. Medved, B. Aukst Margetić, A. Ražić Pavičić, T. Filipec Kanižaj, V. Medved

**Affiliations:** ^1^ University Hospital Center Zagreb; ^2^ Univeristy Hospital Center Sestre milosrdnice; ^3^ Medical School University of Zagreb; ^4^University Hospital Merkur, Zagreb, Croatia

## Abstract

**Introduction:**

Liver transplantation (LT) is a crucial treatment for end-stage alcoholic liver disease, the most common liver disease in developed countries. Personality traits and cognition, a relatively stable characteristics, are known to be significantly associated with quality of life (QoL). However, how they impact QoL in long-term LT survivors is unclear.

**Objectives:**

The study aimed to assess the associations between personality traits and cognition and their impact on the QoL in long-term LT survivors.

**Methods:**

First time LT recipients due to end-stage alcohol liver disease without long-term complications were consecutively included during standard outpatient care. Sociodemographic and clinical data was collected. Personality traits were assessed using 50-item International Personality Item Pool of the Five-factor model (IPIP), cognition using Mini Mental State Examination (MMSE), and QoL using EuroQoL-5D (EQ-5D) questionnaire.

**Results:**

Eighty-three participants were included (mean age 62.9±7.03y, 90.6% male). Median MMSE score was 27±2.00, and median years since LT 5±2.91. Significant positive associations were found between IPIP dimensions Extraversion (B=0.297, p<0.01), Agreeableness (B=0.384, p<0.01), Conscientiousness (B=0.511, p<0.01), and Emotional stability (B=0.432, p<0.01) with EQ-5D visual analogue scale (EQ VAS). IPIP dimension Conscientiousness (B=0.338, p<0.01) and Emotional Stability (B=0. 379, p<0.01) were significantly associated with descriptive dimension of EQ-5D (EQ-5D-3L). MMSE score was significantly associated with QoL (EQ-VAS B=0.291, p<0.01; EQ-5D-3L B=0.283, p<0.05, respectively). However, MMSE score was not shown to be a statistically significant predictor of QoL, whereas Consciousness was a significant predictor of EQ-VAS (β 1.404, t 3.125), and Emotional stability of EQ-5D-3L (β 0.011, t 2.132).

**Conclusions:**

Some personality traits predicted QoL in long-term LT survivors. Therefore, assessment of personality traits should be considered as a part of pre-LT evaluation within a regular psychiatric clearance evaluation.

**Disclosure of Interest:**

S. Medved: None Declared, B. Aukst Margetić: None Declared, A. Ražić Pavičić: None Declared, T. Filipec Kanižaj : None Declared, V. Medved Grant / Research support from: This work was done as a part of the “Genetic Background of End Stage Alcoholic Liver Disease and Liver Transplantation” study supported by the University of Zagreb, Croatia grant 2017 and 2018.

